# A cross-sectional study to investigate motivation for physical activity in a sample of Iranian community-dwelling older adults

**DOI:** 10.34172/hpp.2020.22

**Published:** 2020-03-30

**Authors:** Seyed Alireza Derakhshanrad, Emily Piven, Bahareh Zeynalzadeh Ghoochani

**Affiliations:** ^1^Department of Occupational Therapy, Shiraz University of Medical Sciences, Shiraz, Iran; ^2^Rehabilitation Sciences Research Center, Shiraz University of Medical Sciences, Shiraz, Iran; ^3^University of St. Augustine for Health Sciences, St. Augustine, Florida, USA

**Keywords:** Elderly, Facility access, Environmental barriers, Physical activity

## Abstract

**Background:** On the basis of the Social-Ecological Model, there are assumed to be three sources of motivation – intrapersonal, interpersonal, and community motivation – that prompt older adults to participate in physical activity (PA). These three motivational sources can lead to PA behavior adherence. Little empirical research exists that investigates which motivational source is more influential in older adults’ adherence to PA, thus creating an area of interest for this research.

**Methods:** A cross-sectional study was used to investigate the relationship between levels of PA and different sources of motivation. The convenience sample of 140 community-dwelling older adults, aged 60 and greater, living in Shiraz, Iran agreed to complete self-reported questionnaires,to measure motivation and PA. Five statistical tests were used: Independent-samples t test, one way ANOVA, Pearson correlation coefficient, chi-square, and ordinal regression.

**Results:** Ordinal regression indicated that gender (P = 0.001, CI: 0.523-2.115) and intrapersonal motivation (P < 0.001, CI: 0.038-0.126) were useful predictors of variations in the levels of PA. Compared to males, females engaged in PA with less frequency (P = 0.006). Community motivation decreased with age (r = - 0.213, P < 0.05). There were no significant relationships between age, educational level, health status, and PA (P > 0.05).

**Conclusion:** Interpersonal and community motivation were insignificant factors for PA participation, perhaps due to non-facilitating environment. Future research should be conducted to investigate the environmental issues that hinder PA participation in older adults.

## Introduction

There is a growing body of literature that highlights the positive impact of physical activity (PA) on the healthy aging process.^1^ PA, defined as movements of body produced by the contraction of skeletal muscles that result in energy expenditure increase, is thought to increase active life expectancy and provide older people with physiological, psychological, and cognitive benefits.^2^ PA has been classified into levels, determined mainly by the intensity and time involved in doing the activity, including ‘Inactive’, ‘Insufficiently active’, ‘Active’, and ‘Highly active’. Inactive type is defined as not moving beyond basic movement from daily life activities. Insufficiently active type is defined as doing moderate-to-vigorous intensity PA less than 150 min/wk. Active type is defined as doing moderate-to-vigorous intensity PA between 150 and 300 min/wk. Engaging in more than 300 min/wk of moderate-to-vigorous PA is defined as highly active.^3^ It is recommended that older adults engage in at least 150 min/wk of moderate-to-vigorous intensity PA, such as walking briskly at 2.5 to 4.0 mph.^2,4^ The commonly acknowledged determinant factors that can influence PA behavior adherence include “self-motivation, past activity level/program participation, exercise group cohesion, social support from family, and actual and perceived access to exercise facilities” (p. 204).^5^ On the other hand, physical inactivity is a leading risk factor for death.^6^ Physical inactivity is reported to be high among the adult population. There is a trend in choosing a sedentary lifestyle that progressively increases with age, as patterns of inactive lifestyle habits appear to be embedded in the personalities of older adults,^7^ causing the older population to be generally less physically active than the younger population.^8^

Striving for a more comprehensive understanding of formation of lifestyle habits of older adults’ PA has led to the application of theories and models such as the Self-Determination Theory^9^ and Social-Ecological model,^10^ in order to find determinants of and to categorize the nature of motivators and barriers for older adults’ PA involvement. Each of these theoretical frameworks includes the personal factors (i.e., demographics, cognitive variables) and/or environmental factors (i.e., social and physical), which contribute to the formation of an individual’s lifestyle choice to be an active or a sedentary person. The Social–Ecological model addresses PA by calling attention to both personal and environmental factors.^11^ The model describes how the physical behavior of elderly people is influenced by intrapersonal (personality traits), interpersonal (supports from family or friend), and community (structural/organizational) factors.^12^ The findings of several studies regarding barriers and facilitators of PA in older adults fit well with the Social–Ecological model.^10,13^ Therefore, it has been argued that this model provides a reliable theoretical basis to address PA participation in older adults.^10,13^ Particularly noteworthy in this model is motivation, exhibited thorough an individual’s enthusiasm to initiate and sustain PA, which may be influenced by intrapersonal, interpersonal and community factors.^10^ As a result, the source of motivation to participate in PA can be classified into internal and external factors, known as internal and external motivation.^14^ Internal motivation refers to the desire to participate which is influenced by personal reasons, such as the desire to have more energy. Alternately, external motivation refers to the desire to participate which is influenced by outside reasons, such as a wish to win an award in a competition.^14^ In other words, extrinsic motivation is based on an external benefit from PA such as ‘improving attractiveness’ or ‘feel appreciated’, while intrinsic motivation is based on the internal psychological benefits such as ‘enjoyment’ or ‘improvement of physical or motor competence’.^15^ According to the Self-Determination Theory of Motivation, a theoretical framework for studying PA motivation in older adults, intrinsic motivation would induce older adults to engage in PA for an inherent reason such as interest in or enjoyment of the activity itself, whereas extrinsic motivation is related to outside control or external reasons that include personally-valued outcomes such as fitness, social affiliation, and appearance.^16^ A sample of 725 older adults was found to have multiple, coexisting kinds of motivation for PA, including having fun (internal motivation) as well as medical recommendations (external motivation).^17^

### Model informing this study

The Social–Ecological Model was selected because it was best aligned with the purpose of research that evaluated both personal and environmental factors as determinants of PA. From the perspective of the Social–Ecological model, the source of motivation for PA is further sub-divided into three categories, namely intrapersonal, interpersonal, and community motivation.^12,18^ Intrapersonal motivation refers to an individual’s knowledge, attitudes, beliefs, and personality traits that influence the desire to participate in PA. Interpersonal motivation suggests the positive role of others including family, friends, and peers, on enhancing PA participation. Community motivation includes the affirmative environmental influence of social networks, facilities, rules, norms, and policies that regulate or support participation.^18^ Clearly, intrapersonal motivation on the basis of the Social-Ecological model might be equated with intrinsic motivation proposed by the Self-Determination theory, and interpersonal as well as community motivation, based on the Social-Ecological model, could be equated to extrinsic motivation according to the Self-Determination theory. It is suggested that Self-Determination theory provided considerable flexibility for understanding levels of PA in relation to intrapersonal, interpersonal, and contextual sources of motivation that promote PA participation.^19^

Although considerable research has been devoted to verifying the importance of motivation for participation in PA, the question remains as to which type of motivational source (internal or external) contributes the most to engagement.^16^ In fact, there is a need to investigate how various types of Social-Ecological motivational sources influence older adults’ PA. Most studies published thus far are qualitative and speculative in nature.^13,20^ With quite a small sample, the conclusions of the research were that both personal and environmental factors were motivational determinants that encourage older adults to participate in PA. However, little attention has been paid to the empirical work, in order to determine which source might have the most influential impact. Understanding PA motivational determinants in this way could help healthcare providers identify the critical aspects of motivational sources to design the best interventional approach for encouraging older adults’ participation in PA. Therefore, the purpose of this quantitative study was to identify which source of motivation – intrapersonal, interpersonal, community – might be most influential in determining an older adult’s willingness to engage in PA. Accordingly, our research question was: Does a certain type of motivational source, based on the Social-Ecological model, differentiate PA levels? It was hypothesized that there would be no significant difference among all three motivational sources.

## Materials and Methods

### Study population and sampling

This is a cross-sectional study with predictive purposes,^21^ to investigate how the three motivational sources might determine the levels of PA. This study was conducted between March 2019 and August 2019. A convenience sample was drawn from community-dwelling older adults, who were non-randomly selected from eight local parks across the city of Shiraz, which is a large city in Iran, located in the south of the country. Using statistical software, the minimum required sample size of 140 subjects was calculated to find an estimated correlational coefficient of up to 0.25, where α = 0.05 and β = 0.2.

Eligible subjects who met the inclusion criteria were those with (*a* ) a history of at least 6 months of doing 1 h/wk PA that exceeded basic movements from daily life activities, including outdoor brisk walking, stretching exercises, or playing some sort of sport-related activity (bicycling or swimming); (*b* ) age over 60 years; (*c* ) the ability to walk independently without any assistive device such as cane or crutch; (*d* ) no history of recognized, obvious mental or physical disability; (*e* ) the capacity to understand and complete the research procedure, and (*f* ) Iranian nationality, while residing in the city of Shiraz at the time of the study.

The factor of engaging in ≥1 h/wk PA during the past 6 months helped to confirm that older adults’ PA behavior was habitual and sustained as ‘PA initiation’ and ‘PA maintenance’ (“sustained participation in regular PA for at least six months up to the development of a habit” p.149) are two distinctive issues that should be distinguished in practice and research.^22^ In fact, newly active and long-term regular exercisers are different regarding their motivation for PA.^16^

### Measures

Participants were asked to complete the Participation Motivation Questionnaire of Older Adults in Physical Activity (PMQOA) and a demographic checklist that provided factors such as age, gender, history of chronic disease (i.e., diabetes or hypertension), education, and marital status by self-report.

Asking participants about the frequency of PA during the week has been regarded as one method of subjectively measuring of PA.^23^ Thus, in addition to demographic data, a question was included to gather data about the weekly frequency of doing PA. Participants responded to this item on a 5-point Likert scale (1 h/wk, 2 h/wk, 3 h/wk, 4 h/wk, and ≥5 h/wk).

Corresponding with the Social–Ecological model^11^ that informed this study, the PMQOA is a Persian 35-item self-reported questionnaire that has a three-factor structure to measure participants’ intrapersonal, interpersonal, and community motivation for doing PA.^18^ For example, items such as “I enjoy doing physical activity.”; “My sport coaches encourage me to do physical activity.”; and “The existence of safe places around us encourages me to take part in physical activity.” were three items that assessed intrapersonal, interpersonal, and community motivation, respectively. Participant responses to items were rated on a 5-point Likert scale from “complete agreement” to “complete disagreement”. The scores obtained from each sub-scale, including intrapersonal (15 items with ranges 15-75), interpersonal (8 items with ranges 8-40), and community (12 items with ranges 12-60) were used by the authors for the statistical analysis purposes of this study, where higher scores in each sub-scale identified participants with higher degrees of motivation to participate in PA. Psychometric analysis revealed the high validity and reliability for the questionnaire.^18^ Developers of the PMQOA reported high values of Cronbach’s alpha for three subscales of motivation, including 0.751 (intrapersonal), 0.702 (interpersonal), 0.760 (community), and 0.813 for total scale.^18^

### Procedures

Informed consent was obtained from all participants, who research assistants encountered in the parks. The research assistants approached potential participants in local parks in different parts of the city, in order to recruit a diverse population. After agreeing to participate in the study, the participants were screened to determine if they met the inclusion criteria. About 190 people were excluded as a result of not meeting the inclusion criteria, reluctance to participate, or poor comprehension of the procedures. The participants were provided with the research materials (a clipboard and pen), questionnaires, and instructions for completion. The research assistants were consistently trained to read and complete the questions for those who were unable or unwilling to do it on their own and explain informed consent. All participants gave their informed consent to participate in this study.

### Data analysis 

SPSS software (version 23, IBM Corporation, Armonk, NY, USA) was used to analyze the data. Significance was set at α= 0.05 for each test. Data were expressed by frequencies, means, and standard deviations. The frequency of doing PA was transformed into an ordinal variable called ‘levels of PA’ with three categories titled: (*a* ) ‘Insufficiently active’ with < 3 h/wk PA, (*b* ) ‘Active’ with 3-4 h/wk PA, and (*c* ) ‘Highly active’ with ≥ 5 h/wk PA. All the variables had a normal distribution, so the parametric tests were used to analyze the data. An independent-samples *t* test and one-way ANOVA were used to compare means. The Pearson correlation coefficient was used to determine the relationships among quantitative variables. A chi-square test was used to find the relationships between ordinal (i.e., levels of PA) or nominal (i.e., gender, education, marital status) variables. Ordinal regression was used to determine the contribution of motivational sources to the levels of PA. Gender, chronic disease, educational level, and marital status were entered into the regression model, considered as confounder variables.

## Results

A total of 140 participants (86 male, 54 female) took part in this study. The mean age of the participants was 68.2 (SD 7.1) years (range 60-90). Table 1 presents general characteristics of the study population regarding levels of PA.

As shown in Table 1, the distribution of participants was significantly different among three levels of PA in regard to marital status, gender, and intrapersonal motivation. Gender appeared to be a significant factor in the prediction of PA, with slightly over half of the male participants (51.1%) reported having ≥5 h/wk PA, while slightly over half the female participants (53.7%) reported having 3-4 h/wk PA. It also seems that married participants tended to be more physically active than single participants. Additionally, the more active the participants were, the higher degrees of intrapersonal motivation they had (*P* < 0.001). However, an independent-samples *t* test showed no significant difference between males and females for intrapersonal motivation (*P* = 0.214). Table 2 shows the means, standard deviations and bivariate correlations among the quantitative variables.

The results of the correlation analysis showed that intrapersonal and interpersonal motivation were positively associated with PA (see Table 2). A significant negative correlation coefficient was also found between community motivation and age, indicating that older participants had lower degrees of community motivation.

Table 3 shows the results of Ordinal regression, which indicate that intrapersonal motivation (*P* < 0.001, CI = 0.038-0.126) and gender (*P* = 0.001, CI = 0.523-2.115) were significant predictors of levels of PA.

## Discussion

The purpose of this study was to examine the source of motivation – intrapersonal, interpersonal, community – that would best predict the levels of PA in a sample of Iranian community-dwelling older adults. The findings revealed that intrapersonal motivation and gender could be considered determinants of PA participation in this study population. Specifically, participants who were intrinsically motivated and were male tended to be more physically active than others. There is scant empirical literature about sources of motivation (internal or external) that discriminate levels of PA in older adults.^16^ In terms of gender differences, a body of research shows that women are less likely to participate in PA than men. For example, in a quantitative study about older adults, also comprised of data from self-administered questionnaires, women were found to be less physically active than men, due to personal and environmental restrictions related to PA.^24^ The authors’ results are also in line with an Iranian quantitative study that reported a significant relationship between PA, marital status, and gender, but in contrast with our study, that study found significant relationships between PA, age, educational level, and health status,^25^ perhaps due to the difference between demographic characteristics of the two samples. Another cause of this inconsistency may be related to the fact that this study included a sample of physically active older adults who were found in parks. Since the participants were encountered in a park, it could be assumed that they had more positive thoughts about the necessity to be physically active,^26^ regardless of their age and other demographic background factors.

The results from the ordinal regression indicated that gender and intrapersonal motivation could predict variations in the levels of PA. This means that interpersonal and community motivation failed to differentiate the levels of PA. According to our hypothesis, this study population could have also been influenced by other people as motivators (interpersonal motivation) or structural/organizational factors (community motivation). However, it was an intrapersonal factor that differentiated the levels of PA. It is difficult to explain this result, but the result might be related to the premise that participants perceived others and surrounding factors as not encouraging for PA. It is possible that the participants’ family members, such as a spouse or partner, may have been physically inactive themselves, thus undermining participant motivation, or the participants were surrounded by a non-facilitating environment with less motivational attributes for PA participation. Unlike our study, Molloy et al reported social environment to be a potential source for promoting PA participation by triggering interpersonal and community motivation, especially among women.^27^ In particular, the external influence of environmental factors that facilitated interpersonal and community motivation were perceived as insignificant, which might explain why female participants were physically less active than men. Because women were actually reported to be particularly prone to extrinsic motivation for PA,^28^ it may be possible that female participants have encountered greater difficulties in doing PA in their environment due to a lack of support, safety concerns, and scenery.^24^

Interpersonal and community motivation might be ranked insignificant in this study population because the participants might have not been offered substantial incentives from external factors, such as encouragement by others, or access to safe facilities, which were considered to be two important environmental factors that provided extrinsic motivation for PA in a group of active American older adults.^29^ The results indicated in Table 2 may provide evidence for this claim because the participants’ community motivation decreased with age (r = -0.213, *P* < 0.05), indicating possible perceived barriers posed by the non-facilitating environment as people age.^30^

Another possible explanation for the findings of this study is related to the characteristics of the study population sampled for this research. Since participants’ PA became habitual by ≥6 months, they seemed to be more intrinsically motivated, as this type of motivation from within could generate commitment to PA.^16^ In accordance with the present results, Dacey et al’s study has demonstrated that intrinsic motivation could be one of the main determinants that was positively associated with the increased PA that was sustained in older adults.^16^ This inference also seems to be in accordance with that of Navarro et al, who reported that older adults’ PA participation following outside control (i.e., medical advice) did not stem from the internal perception of how much benefit PA could bring, as opposed to intrinsic factors.^15^ It could be argued that this study population might have engaged in PA because they sought some sort of internal benefit from PA, such as enjoyment or satisfaction. It is suggested that extrinsic motivation that stems from the environment can help older adults start PA, while intrinsic motivation is the key to success or long-term commitment to PA.^31^

Future research about environmental issues is warranted because socio-cultural and environmental barriers such as air pollution and lack of safe public places, which can lead to fear of accident and injury, were acknowledged to have contributed to a decrease in older adults’ PA in a sample of the Iranian population.^32^ In terms of environmental issues, our study may also confirm the findings of another Iranian qualitative research study that explained how lack of autonomy (restrictions posed by others), lack of environmental safety, poor public transport, and inaccessibility to sport facilities played roles as motivational barriers to older adults’ engagement in PA.^33^ With a focus on education and motivation, the findings of this study suggest developing useful strategies that can be implemented by communities to improve external factors and empower older adults’ interpersonal and community motivation for PA. Some strategies are providing free access to group-based exercise classes at senior facilities, raising awareness of the benefits of PA, and providing opportunities for social interaction through the construction of open spaces and attractive walking areas.^31^

### Limitations

The main limitation of this research is that it was a cross-sectional study, using self-reported data and a self-administered questionnaire, which made it difficult to infer and discuss causal relationships between variables, especially in regard to the insignificant correlations between PA, age, educational level, and health status. Moreover, participants’ PA levels were measured subjectively, not objectively by participants themselves. Another limitation was related to the selection of a non-random convenience sample, in a place where people typically go to exercise. Apart from these limitations, this research findings showed there might be some environmental issues that hampered our participants’ aptitude to rank interpersonal and community motivation as influential motivational sources for PA participation.

## Conclusion

This study has identified intrapersonal motivation as the main source for PA participation among the study population. In contrast to earlier findings, no evidence of external influence (environmental factors) was detected to be significant for developing interpersonal and community motivation. It is likely that participants perceived environmental factors as not too encouraging for participation in PA. Future research should examine the environmental issues to find out why environmental factors played an insignificant role in encouraging this population to engage in PA. Future research should also investigate the type of supports that might create a more conducive, encouraging environment that attracts older adults to engage in PA.

## Ethical approval

The present study was approved by the ethics committee of Shiraz School of Rehabilitation Sciences affiliated with Shiraz University of Medical Sciences, Shiraz, Iran (Approval ID: IR.SUMS.REHAB.REC.1397.009).

## Competing interests

The authors report no competing interest.

## Funding

This research was financially supported by Rehabilitation Sciences Research Center, Shiraz, Iran (Grant number: 97-01-51-18295).

## Authors’ contributions

All authors made contributions to the study conception and design. Data analysis was done by SAD. All authors made contributions to the interpretation of data. In addition, they also contributed to the drafting and revision of the article as well as approval and submission of the final version.

## Acknowledgments

We would like to thank the cooperation of participants and the financial support of Rehabilitation Sciences Research Center.

**Table 1:** General characteristics of the study population regarding levels of physical activity

**Variables**		**Insufficiently active (n=24)**	**Active (n=59)**	**Highly active (n=57)**	***P*** **value**
Age		66.6 ± 7.5^a^	69.7 ± 8.3	67.04 ± 5.5	0.068^c^
60-70		19 (19%)^b^	37 (37%)	44 (44%)	
71-80		3 (9.7%)	16 (51.6%)	12 (38.7%)	0.188^d^
81-90		2 (22.2%)	6 (66.7%)	1 (11.1%)	
Intrapersonal motivation		53.8 ± 12.2	59.2 ± 10.2	63.7 ± 7.8	<0.001^c^
Interpersonal motivation		23.4 ± 7.3	24.5 ± 7.8	25.9 ± 6.5	0.314^c^
Community motivation		31.0 ± 9.8	33.9 ± 11.04	34.8 ± 10.6	0.340^c^
Gender	Male	12 (14%)	30 (34.9%)	44 (51.1%)	0.006^d^
	Female	12 (22.2%)	29 (53.7%)	13 (24.1%)	
Chronic disease	Yes	12 (21.4%)	28 (50%)	16 (28.6%)	0.067^d^
	No	12 (14.5%)	31 (37.3%)	40 (48.2%)	
Education	Up to diploma	14 (15.1%)	39 (41.9%)	40 (43.0%)	0.587^d^
	College	10 (21.3%)	20 (42.6%)	17 (36.1%)	
Marital Status	Single	1 (3.8%)	18 (69.2%)	7 (27.0%)	0.006^d^
	Married	23 (20.2%)	41 (36.0%)	50 (43.8%)	

^a^ Mean ± SD; ^b^ No. (%); ^c^One-way ANOVA; ^d^ Chi-square test.

**Table 2:** Means, standard deviations (SD), and bivariate correlations among quantitative variables (N=140)

**Measures**	**Mean**	**SD**	**1**	**2**	**3**	**4**
1. Age	68.2	7.1	-			
2. Intrapersonal motivation	60.1	10.2	-0.049	-		
3. Interpersonal motivation	24.8	7.2	-0.086	0.609**	-	
4. Community motivation	33.8	10.7	-0.213*	0.447**	0.722**	-
5. Physical activity	3.8	1.3	0.017	0.376**	0.170*	0.129

* *P* < 0.05, ** *P* < 0.01.

**Table 3:** Ordinal regression of variables predicting levels of physical activity

**Variables**	**Estimate**	**(95%) CI**	***P*** **value**
Age	-0.032	-0.084, 0.019	0.217
Intrapersonal motivation	0.082	0.038, 0.126	0.000
Interpersonal motivation	-0.053	-0.130, 0.025	0.185
Community motivation	-0.007	-0.054, 0.041	0.789
Gender			
Male	1.319	0.523, 2.115	0.001
Female	Referent	-	-
Chronic disease			
Yes	-0.409	-1.130, 0.313	0.267
No	Referent	-	-
Education			
Up to diploma	0.425	-0.298, 1.149	0.249
College	Referent	-	-
Marital status			
Single	0.691	-0.254, 1.635	0.152
Married	Referent	-	-
